# Suicidal patients’ experiences regarding their safety during psychiatric in-patient care: a systematic review of qualitative studies

**DOI:** 10.1186/s12913-017-2023-8

**Published:** 2017-01-23

**Authors:** Siv Hilde Berg, Kristine Rørtveit, Karina Aase

**Affiliations:** 1 Division of Psychiatry, Stavanger University Hospital, N-4068 Stavanger, Norway; 2 Department of Health Studies, University of Stavanger, N-4036 Stavanger, Norway

**Keywords:** Patient experiences, Patient perspective, Mental health, Psychiatric care, In-patient, Suicidal, Suicide, Patient safety

## Abstract

**Background:**

In-patient suicide prevention is a high priority in many countries, but its practice remains poorly understood. Patients in a suicidal crisis who receive psychiatric care can provide valuable insight into understanding and improving patient safety. The aim of this paper was therefore to summarize the qualitative literature regarding suicidal patients’ in-patient care experiences. The following question guided the review: How can we describe suicidal patients’ experiences regarding safety during psychiatric in-patient care?

**Methods:**

Systematic searches were conducted in the MEDLINE, Academic Search Premier, CINAHL, SOCINDEX and PsycINFO databases, identifying 20 qualitative studies on suicidal patients and their psychiatric in-patient care experiences. These studies were systematically reviewed using the Preferred Reporting Items for Systematic Reviews and Meta-Analyses (PRISMA) guidelines, synthesized via thematic analysis and subjected to quality appraisals.

**Results:**

Patients described safety as “feeling safe”, and three components, i.e., connection, protection and control, were vital to their experiences of safety. Fulfilling these needs was essential to patients recovering from suicidal crises, feeling safe during encounters with health care professionals and feeling safe from suicidal impulses. Unmet needs for connection, protection and control left patients feeling unsafe and increased their suicidal behaviour.

**Conclusion:**

Our review addresses the importance of adopting a wider perspective of patient safety than considering safety solely in technical and physical terms. Safety for the suicidal patient is highly dependent on patients’ perceptions of their psychological safety and the fulfilment of their needs. The three patient-identified factors mentioned above – connection, protection and control – should be considered an integral part of patient safety practices and should form the basis of future efforts to understand the safety of suicidal patients during psychiatric in-patient care.

**Electronic supplementary material:**

The online version of this article (doi:10.1186/s12913-017-2023-8) contains supplementary material, which is available to authorized users.

## Background

Suicide is a particular concern in mental health settings because of its strong association with mental illness [1]. Although suicides rarely occur during in-patient care, these events are clinically important and are among the most concerning patient safety incidents in the mental health sector [2–4]. Suicide prevention is one of the primary tasks of health care professionals practicing in psychiatric wards [4]. In-patient suicide prevention is a high-priority in many countries [5–7]; however, its practice remains poorly understood.

The ethical and pragmatic problems posed by including suicidal patients in research have contributed to the currently limited research regarding the treatment of high-risk and hospitalized suicidal patients [8]. To understand safety in health care services, information must be obtained from multiple sources, including the patient’s perspective. As such, patients can provide insight regarding care and can contribute important information when other sources of evidence are limited [9]. Patients can also provide unique information on adverse events in hospitals [10, 11] as well as useful descriptive feedback regarding safety, in particular sensitive safety-related topics [12]. Patient experiences are considered one of the three pillars of health care quality, along with clinical safety and effectiveness of outcomes [13].

Qualitative studies of patient experiences with psychiatric in-patient care have been reviewed within certain areas, such as involuntary hospitalizations [14], physical restraint [15], acute wards [16], seclusion practices [17], locked doors [18] and service user expectations [19]. However, no reviews to date have examined studies regarding suicidal in-patients. Therefore, this review aimed to summarize empirical qualitative studies by exploring suicidal patients’ psychiatric in-patient care experiences to better understand their perspectives toward safety.

### Review question

A literature review was conducted to answer the following review question: How can we describe suicidal patients’ experiences regarding safety during psychiatric in-patient care?

## Methods

The selected studies were systematically reviewed using the Preferred Reporting Items for Systematic Reviews and Meta-Analyses (PRISMA) guidelines [20]; the articles were then synthesized using thematic analysis [21] and assessed further via quality appraisal [22]. The objectives, inclusion criteria, analysis methods and search strategy were specified and documented in a protocol reviewed by the three authors prior to the database search. The authors are researchers with backgrounds in psychology (SHB), mental health nursing (KR) and safety science (SHB and KAA).

### Inclusion and exclusion criteria

The eligibility criteria for inclusion in the review pertained to the following three characteristics: *Type of study*: Qualitative peer-reviewed studies in English with empirical data on patients’ experiences regarding safety were eligible. *Participants*: Studies examining a sample of suicidal in-patients who were interviewed during their hospitalizations or after discharge were eligible. “Suicidal in-patients” included patients hospitalized after a recent suicide attempt, described as suicidal during hospitalization or with serious suicidal thoughts or ideations; self-harming behaviour was excluded. The final criteria related to *Setting:* Experiences regarding care in psychiatric hospital wards, including psychiatric emergency wards and psychiatric long-term in-patient care, were eligible. Studies in multiple hospital settings were included if information regarding psychiatric in-patient care experiences could be extracted. Patient experiences pertaining to outpatient clinics, community mental health care, home care, forensic psychiatric services, emergency care and medical care were excluded. Studies describing patient experiences with adverse side effects from pharmacological treatment were excluded. Studies with mixed patient samples and studies involving health care professionals’ experiences were included if information regarding patient experiences could be extracted.

### Search strategy and study selection

To increase sensitivity, limitations on publication date were not imposed during the database search. The selection of databases, search terms and search methodology were determined in collaboration with a university librarian. The databases included in the systematic search were MEDLINE and the Academic Search Premier, CINAHL, SocINDEX with Full-Text and PsycINFO Ovid databases. Systematic database searches were conducted between June and December 2014 and in July 2016.

Search terms were identified in relevant studies during the planning of the systematic review. The terms were selected from qualitative studies of patient experiences in mental health care and from qualitative studies of suicidal patients’ experiences. All identified search terms were included to increase search sensitivity. The full electronic search strategy for PsycINFO is outlined in Additional file 1. We also screened reference lists and conducted author searches in EMBASE and Google Scholar.

We systematically searched all of the above databases using the following terms: patient* satisfaction*, patient* preference*, in-patient* experience*, patient* experience*, patient* perception*, patient* view*, patient* perspective*, patient* opinion*, user* experience*, consumer* experience*, consumer participation, suicide, suicidal, feeling safe and feeling unsafe.

**Fig. 1:**
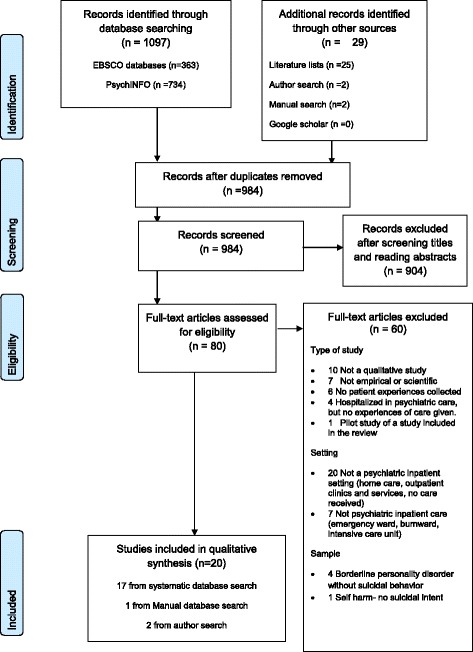
PRISMA (2009) flow diagram

The study selection process was conducted according to the eligibility criteria displayed in the flow diagram in Fig. 1. First, all titles were screened, and the abstracts were read by one author (SHB). Ineligible studies were excluded. Full-text articles were obtained for the eligible studies. Two authors (SHB and KR) independently assessed the full-text articles for eligibility in a standardized manner. A third author (KAA) validated the assessments. The level of agreement was generally high; however, setting was often discussed, as the studies were conducted in mixed settings. Agreement was reached by re-reading the articles to determine whether information on patient experiences with psychiatric in-patient care could be extracted from the studies in question. All authors were in agreement regarding the final inclusion and exclusion of all articles. A data extraction sheet was developed to guide study selection. Information from all full-text articles was added to the sheet. All studies were assessed based on the abovementioned eligibility criteria and colour-coded as red (no), orange (maybe) or green (yes).

### Synthesis of results

Thematic analysis, as proposed by Thomas and Harden [21] and Braun and Clarke [23], was used to facilitate the synthesis of the results of the included studies. The thematic synthesis consisted of two stages. The first stage entailed coding the text “line by line”, condensing the meaning units and developing descriptive themes. An inductive approach was used in which the descriptive themes remained close to the original findings of the studies [23]. The second stage developed relationships between the descriptive themes and patient safety to generate analytical themes [21]. Thematic mapping was used to identify relationships between meaning units, descriptive themes and analytical themes [21, 23]. Connections between patients’ needs, expectations, experiences, reported outcomes (such as experiencing increased or decreased suicidal behaviour) and use of the term ‘safety’ were studied in the analytical stage. Coding and preliminary theme development were conducted by one author (SHB) and reviewed by all three authors. The analysis yielded 83 meaning units, nine descriptive themes and three analytical themes (“Connection”, “Protection” and “Control”). Forty-nine of the 83 meaning units were found in the “Connection” theme, which was thus considered the most comprehensive theme. An example of theme condensation is presented in Table 1. A full overview of the meaning units and themes is provided in Additional file 2.

**Table 1:** Example of theme condensation

Example of extracted data	Meaning units	Descriptive theme	Analytical theme
Lack of acknowledgment from observers; these perceptions sometimes overlapped with perceptions of a lack of empathy. Such behaviors included observers’ reading books, appearing distracted or uninterested in the participant, and acting like the participant was a burden [40].	61. Lack of observer support manifests as lack of empathy and acknowledgement	Receiving support from the observers	Protection
Feelings of objectifications in formal observation without interpersonal engagement…It’s a scary thing going somewhere where you feel like you’re isolated and locked away. (Claire)…Being watched like that; it’s freaky…a bit invasive…that separation, that ‘us and them’. It’s a bit tricky. (Kate) [26].	62. Feeling objectified and detached without observer support		
“They don’t care. You get that feeling quite often. It just kind of supports that hopeless kind of feeling that life isn’t worth living and nobody cares about anything.” Such encounters did little to alleviate hopelessness, and six participants noted that they increased their anxiety or aggravated their dysphoria [40].	63. Feeling objectified increases stress and hopelessness		

Two authors (SHB and KR) independently assessed the methodological quality of the included studies and rated the studies based on Malterud’s [22] checklist for qualitative research. Malterud’s guidelines for assessing qualitative studies and an example of a scored article are provided in Additional file 3. An overview of the quality assessment of the included papers is presented in Additional file 4.

## Results

### Study selection

The study selection process utilized the PRISMA guidelines [20] (Fig. 1) and identified a total of 1,097 records through database searches. Additional searches yielded 29 records. After removing duplicates, the remaining 984 records were screened. Title screening and abstract reading resulted in the exclusion of 904 records that did not meet the eligibility criteria. Eighty full-text articles were read, and relevant information was extracted and entered into the information sheet, assessed according to the inclusion criteria and coded (yes/maybe/no). Sixty records were excluded for not meeting the inclusion criteria, and we ultimately included the remaining 20 studies in the systematic review (Table 2).

**Table 2:** The list of included studies

Author/year/origin	Aim	Sample	Setting	Data collection methods and analysis	Key points related to in-patient care
Vatne & Nåden, 2016 [38]. Norway	To develop a deeper understanding of suicidal patients in the aftermath of suicide attempts	Ten patients considered with serious suicidality after a suicide attempt. Non-psychotic. Interviewed after suicide attempt.	Two emergency psychiatric wards and one crisis resolution team.	Semi-structured interviews. Analysed using thematic analysis inspired by Braun and Clarke. Gadamerian hermeneutic approach.	• Connectedness, someone who cares • Hospital admission important for staying alive • Support from family and friends
Lees, Procter and Fassett, 2014 [26]. Australia	To explore the experiences and needs of mental health-care consumers who had a suicidal crisis (shortened).	Nine patients recovered from a recent suicidal crisis where they received mental health in-patient care.	Setting not specified. Experiences of psychiatric in-patient care are described.	In-depth, semi-structured interviews collected as part of a larger multi-method study. Analysed with a constant comparative method and classical content analysis.	• Therapeutic engagement central to quality of care • Isolation, loss of control, objectification
Montross Thomas et al., 2014 [28]. USA	To better understand suicide experiences from the perspective of patients diagnosed with serious mental illness.	23 patients hospitalized after a suicide attempt. Diagnosed with serious mental illness. Interviewed after discharge.	Veterans Affairs Hospital, mental health program.	Qualitative interviews with audio/videotaping. Analysed using van Manen’s phenomenological framework.	• Need for clinicians’ empathy, compassion and listening skills • Addressing problems underlying suicide attempt
Vatne & Nåden, 2014 [32]. Norway	To explore the experiences of being suicidal and encounters with health care personnel.	Ten patients considered seriously suicidal. Psychosis excluded. Interviewed after suicide attempt.	Psychiatric emergency ward, sub-emergency psychiatric wards and one crisis resolution team.	Semi-structured interviews. Analysed using thematic analysis inspired by Braun and Clarke. Gadamerian hermeneutic approach.	• Openness and trust • Someone who addresses the matter • Being met on equal terms, humiliated
Cutcliffe et al., 2012a [41]. Unknown origin.	To better understand the observed increased risk for suicide following discharge from an in-patient psychiatric service. Key theme one.	20 patients admitted to the hospital with suicidal ideation and/or a lifetime history of suicidal behaviour. Interviewed after discharge.	In-patient psychiatric service.	Hermeneutic interviews. Analysed using van Manen’s phenomenology.	• Anxiety to go back to life without having a sense of control • Need to be involved in discharge planning
Cutcliffe et al., 2012b [42]. Unknown origin.	To better understand the observed increased risk for suicide following discharge from an in-patient psychiatric service. Key theme two.	20 patients admitted to the hospital with suicidal ideation and/or a lifetime history of suicidal behaviour. Interviewed after discharge.	In-patient psychiatric service.	Hermeneutic interviews. Analysed using van Manen’s phenomenology.	• Patients still suicidal at discharge • Disorientation concerning what to do with their life • Need for post-discharge support
Pavulans et al. 2012 [27]. Sweden	To explore the experience of being suicidal, including a suicide attempt, and identify possible implications for health care professionals.	Ten patients interviewed after a suicide attempt while hospitalized in a psychiatric ward.	Psychiatric in-patient care at one university hospital.	Semi-structured interviews. Analysed using van Manen’s phenomenology and qualitative content analysis.	• Being in need of control • Re-establish control before the point of no return • Control related to problem-solving and insight
Vatne & Nåden, 2012 [29]. Norway	To explore experiences of persons after a suicide crisis or a recent suicide attempt.	Ten patients considered seriously suicidal. Psychosis excluded. Interviewed after suicide attempt.	Psychiatric emergency ward, sub-emergency psychiatric wards and one crisis resolution team.	Qualitative interviews. Analysed using thematic analysis. Gadamerian hermeneutic approach.	• Losing touch with the world • Someone to see, listen and understand • Desperation increases with involuntary hospitalization
Holm & Severinsson, 2011 [31]. Norway	To explore how recovery processes facilitate changes in suicidal behaviour in women with borderline personality disorder.	13 patients with suicidal behaviour. Borderline personality disorder.	Recruited from different settings within mental health. Experiences of psychiatric in-patient care were described.	In-depth interviews. Data analysed with thematic analysis.	• *Changing suicidal behavior by feeling confirmed, safe, and trusted.*
Cutcliffe et al, 2006 [36]. England	To determine if psychiatric/mental health nurses provide meaningful caring responses to suicidal people, and if so, how was it achieved.	20 patients with experiences from a serious suicide attempt.	Crisis care in emergency psychiatric services.	Semi-structured interview. Data analysed with constant comparative method. Glaserian grounded theory approach.	• Reconnecting the person with humanity • Guiding the individual back to humanity, learning to live
Sun, et al 2006b [25]. Taiwan	Presentation of a nursing care theory developed to guide the care given to people with suicidal ideas and those with a previous suicide attempt.	15 patients with either suicidal ideas or attempted suicide. Interviewed while hospitalized.	Psychiatric hospital ward.	Semi-structured interviews and participant observation. A grounded theory approach.	• Safe and compassionate care giving via the therapeutic relationship
Sun et al, 2006a [24]. Taiwan	To investigate nurses’ and patients’ perceptions of psychiatric wards (the context of care) and the professionals’ response (the intervening conditions) that may impact the delivery of suicidal nursing care.	15 patients with either suicidal ideas or attempted suicide. Interviewed while hospitalized.	Psychiatric hospital ward.	Semi-structured interviews and participant observation. A grounded theory approach.	• Protective environment • Access to lethal items • Group support, spiritual support
Talseth, Gilje & Nordberg, 2003 [30]. Norway	To describe a process of consolation revealed by two suicidal patients’ experiences.	Two patients. Interviewed after a suicide attempt (from the Talseth et al., 1999 [34] study).	Psychiatric hospital ward.	Qualitative interviews. Phenomenological hermeneutic study inspired by Ricoeur’s philosophy.	• Vulnerability and deep despair • Closeness • Connection • The dialogue with HCPs
Wiklander, Samuelsson, & Åsberg, 2003 [33]. Sweden	To extract and analyse the interview data concerning experiences of shame.	13 patients with experiences from attempted suicide. Interviewed after discharge.	Specialized psychiatric in-patient care.	Qualitative semi-structured interviews. Transcripts analysed using qualitative methods (not specified).	• Sensitive to attitudes and behaviours of HCPs • Shame reactions related to aspects of care
Talseth, Jacobsson & Nordberg, 2001 [39]. Norway	To illuminate the experience of being treated by physicians.	21 patients expressing the wish to die or attempted to commit suicide. Interviewed while hospitalized.	Psychiatric emergency wards, psychiatric sub-emergency wards and one psycho-geriatric ward.	Qualitative interviews interpreted using a phenomenological hermeneutic approach inspired by Ricoeur’s philosophy.	• Need for confirmation in interactions with physicians
Samuelsson et al., 2000 [35]. Sweden	To describe the attempted suicide patients’ perceptions of receiving specialized in-patient psychiatric care.	18 patients. Interviewed after a suicide attempt near the time of discharge.	Specialized psychiatric in-patient care.	Qualitative interviews. Analysed for qualitative content using methods inspired by Burnard.	• Perception of care and caregivers, a sense of security • Confirmation and lack of confirmation • Commitment and respect
Cardell & Pitula, 1999 [40]. USA	To explore patients’ experience of constant observation to determine whether they derived any therapeutic benefits beyond the intended protective benefit.	20 patients placed under constant observation for suicidality.	Psychiatric hospital ward and a general medical centre with a psychiatric in-patient unit.	Extensive in-depth interviews. Analysis of themes consistent with Hutchinson’s recommended management of grounded theory data.	• Constant observation not merely a protective intervention, but with therapeutic potential. • Need for engaged and supportive observers
Fletcher, 1999 [43]. UK	To explore the perceptions of staff regarding the constant observation of a suicidal patient in mental health settings.	24 patients at risk for suicide, constantly observed for at least 48 h.	Acute psychiatric hospital.	Ethnographic study with participant observation and semi-structured interviews. Data transcribed onto cards and subjected to content analysis.	• Patients’ negative feelings of being under constant observation related to staff actions
McLaughlin, 1999 [37]. UK	To explore psychiatric nurses’ and patients’ opinions regarding the care offered to suicidal patients and how the care for suicidal patients could be improved.	17 patients admitted for depression, suicidal ideation or overt suicidal behaviour.	Three psychiatric hospital wards.	Observation and semi-structured interview. Data analysed using content analysis by Field and Morse.	• The need to address difficulties • Help with problem-solving
Talseth et al., 1999 [34]. Norway	To illuminate the meaning of suicidal psychiatric in-patients’ experiences of being cared for by mental health nurses.	21 patients admitted with suicidal ideations or after a suicide attempt.	Psychiatric emergency wards, psychiatric sub-emergency wards and one psycho-geriatric ward.	Qualitative narrative interviews. A phenomenological–hermeneutic method inspired by Ricoeur used in the data analysis.	• Being confirmed • Lack of confirmation

Abbreviations: *HCP* health care professional

### Study characteristics

The review consisted of 20 articles published between 1999 and 2016. The patients’ ages ranged from 16 to 63 years. The most frequently occurring diagnoses in the sample were affective disorders, of which major depression was the most prevalent, followed by schizophrenia spectrum diagnoses and personality disorders. Patients reported different experiences and needs depending on their symptoms and level of functioning; however, these parameters could not be analysed because of the presence of mixed samples. All patients had experienced suicidal crises, and the majority had attempted suicide prior to hospitalization. The studies originated primarily from Western mental health care settings, with the exception of studies by Sun et al. [24, 25], which were conducted in Taiwan.

### Themes representing patients’ experiences regarding safety

The results of the 20 studies were synthesized and organized under analytical and descriptive themes (Table 3). The results of this synthesis are described in greater detail in the following text.

**Table 3:** Analytical and descriptive themes

Analytical theme	Descriptive theme
Connection	Meeting someone who cares Receiving a confirmation of feelings Being acknowledged as a human being
Protection	Being protected from death Receiving support from the observers
Control	Gaining insight Coping with difficulties and symptoms Attaining discharge readiness

#### Connection

The “Connection” theme illustrates how connections with health care professionals were vital for patient recovery and feelings of safety. A lack of connection was also experienced by the patients and had potentially fatal consequences. The sample of suicidal patients included in this review reported multiple and diverse causes of their suicidal crises [26, 27], but all patients experienced feelings of overwhelming suffering and increased vulnerability [27–31]. Patients experienced increased emotional sensitivity regarding how they were perceived and approached by health care professionals, and this sensitivity affected their perceptions of themselves, their recent suicide attempt, their therapeutic relationships [26, 32, 33] and their feelings of safety in the hospital [31, 34, 35]. Patients’ connections with health care professionals enabled them to feel valued as human beings by *meeting someone who cares*; to feel understood by *receiving a confirmation of feelings;* and to feel respected and trusted by *being acknowledged as a human being.*

### Meeting someone who cares

Suicidal patients expressed feeling lonely, being alone with their despair, being separated from the external world and feeling a need to be connected with others [28–30, 34]. A sense of being cared for could be achieved by meeting the patient’s basic needs, such as bodily contact, fresh air, food, hygiene, sleep and rest [34]. Patients also felt cared for when they engaged with health care professionals who were active and empathetic listeners, who spent time with them, and who showed interest in them as well as compassion for their situation [26, 28, 34, 36–38]. These interpersonal interactions and the physical presence of the health care professionals helped patients feel that they were valuable [30, 34, 39] and that they mattered and belonged in the world [30, 36]; these feelings reduced their suicidal ideations [36] and made them feel safe in the psychiatric ward [34, 35]. Cutcliffe ([36], s. 797) described this recovery process as a “re-connection with humanity” driven by connecting with and feeling cared for by nurses.

Some patients felt that their health care providers had neither time nor compassion for them [25, 34, 37], and these feelings had potentially fatal consequences. These patients experienced that their health care providers spent little time with them because the providers were busy performing other tasks or were interrupted during patient visits. Some patients experienced having no one to talk to, feeling ignored or feeling that they were being stored away as though they were an object [34, 39]. When met with a lack of interest and disengagement from health care professionals, patients lost confidence in their providers [34], refrained from seeking help and felt unsafe in the ward [35]. The experience of being isolated and alone on the ward raised feelings of hopelessness and worthlessness [39]. Some patients felt redundant and started to plan ways to take their lives on the ward [34].

### Receiving a confirmation of feelings

Patients indicated that they needed someone who could listen to and understand their story and situation [29, 32, 34] and provide confirmation of their feelings [24, 34, 36]. They also expressed a need to be taken seriously in their suffering, to be allowed to express their feelings [33–35] and to be able to talk about their suicidality [28, 32]. The patients positively described their experiences being asked directly about their suicidal thoughts and plans, as they longed for opportunities to talk about difficult questions [32]. Patients felt confirmed when they perceived that their mental health providers understood their situation and their need to step away from the demands of their lives [33] and supported their need for hospitalization [35]. The quality of the patient-physician relationship depended on patients’ experience of this confirmation, as it enabled them to feel safe and understood [34, 36] and mitigated the despair and shame elicited by their suicide attempts [30, 33, 34].

Patients experienced a lack of confirmation when health care professionals denied their feelings, neglected their illness, diverged from topics that the patients wanted to address, did not address difficult feelings [33–35], merely emphasized their positive resources [32], or did not provide adequate or empathetic responses when they disclosed sensitive issues [33]. Some patients reported that their health care professionals did not spend sufficient time with them to properly understand the reasons for their suicide attempts or that the professionals avoided talking about their suicide attempt [34, 39]. Other patients felt that their nurses were concerned only about their symptoms or the effects of their medications and thus did not allow them opportunities to share their thoughts and feelings [34, 39]. Patients perceiving these types of non-responsive attitudes with respect to sensitive or important topics experienced worsening feelings of shame and humiliation [32, 33] that exacerbated their suicidal ideations and, in some cases, resulted in subsequent suicide attempts [32, 35].

### Being acknowledged as a human being

Patients stated that it was important for providers to meet them on equal ground in order for them to feel acknowledged as a human being [26, 33, 34]. This meant being treated non-judgementally [24, 28, 33, 36] – being empowered and understood as individuals rather than as objects, cases or diagnoses [30, 31, 33]. When the patients felt that they were achnowledged as a human being, they were able to feel trusted, respected, and safe in the ward and were thus receptive to help [26, 30, 31, 35]. Through these feelings, patients regained their sense of human dignity and thereby felt that it was worthwhile to be alive [26, 33, 36].

Not being seen as a human being was related to feelings of inequality [32, 34], e.g., patients whose providers overused medical jargon or limited their visits to discussions about medications and diagnoses [31, 34], as well as the feeling of being punished by health care professionals through the use of ward rules, verbal expressions or body language to exert their power [33]. Not being seen as a human being was also related to feelings of disempowerment, e.g., being talked about when they were present [32], not being informed about ward routines [33] or who their primary nurse was [25, 37], not being informed about their own arrangements [35], or experiencing that their opinions, information or histories were not considered important [32, 39]. Suicidal patients with borderline personality disorder experienced that they were able to recover by experiencing feelings of safety and trust during their encounters with health care professionals. However, when treated as inferior, the patients did not feel safe in the hospital [31].

#### Protection

The “Protection” theme pertained to patients’ experiences when under constant observation and their struggles to feel safe from themselves and their invasive suicidal impulses [31, 40]. Patients felt safe from themselves and their suicidal impulses and *protected from death* during constant observation. *Receiving support from the observers* was the most important aspect during constant observation, as patients lacking these relationships felt detached and objectified, and their anxiety and symptoms worsened [26, 40].

### Being protected from death

During constant observation, some patients experienced a state of mind in which they continually searched for available means to attempt suicide. Some experienced feeling powerless against their suicidal thoughts, whereas others experienced command hallucinations related to suicide [40]. Patients perceived constant observation as a means of altering their suicidal ideations and self-destructive behaviour. Patients considered this practice life-saving because of the presence of vigilant observers, the limited availability of objects to use for suicide attempts, the passage of time [40] and the distraction and escape from the outside world [24]. Patients struggled to feel safe from themselves and to assume responsibility for their own lives when they lacked protection during acute suicidal crises [31, 41]. Adequate protection was also related to their perceptions of the hospital as a safe place [41, 42]. Accordingly, patients who easily found ways to attempt suicide in the ward and those who did not receive safety searches or monitoring often felt unsafe in the hospital [25].

However, one patient explained that not being able to end his life actually increased his suffering, as he believed that being able to end his suffering in the event that it became unbearable was a source of comfort that helped him cope with his situation [29]. Patients experienced a lack of freedom and privacy under constant observation [25, 40, 41], and most were happy when it was discontinued because of its invasiveness. Some patients even lied about their suicidality to discontinue their observation [40].

### Receiving support from observers

Cardell and Pitula [40] concluded that the relationship with care providers was at the heart of constant observation and highlighted the importance of patients having supportive observers as opposed to impersonal and detached observers. Patients experienced observer support as vital for decreasing their suicidality during constant observation [36, 40], as these relationships facilitated reduced suicidality. It was important for the observers to have an optimistic attitude, encourage problem-solving, enable patients to gain self-esteem, acknowledge patients as unique and meaningful human beings [40], and try to understand patients by talking with them about their feelings [43]. By interacting with supportive observers, the patients internalized what the observers projected and felt worthy as human beings and thus worthy of being alive [40].

Some patients experienced a lack of acknowledgement and a lack of interpersonal engagement under constant observation, in which the observers appeared disinterested or distant or behaved as though their patients were a burden [26, 40]. When attempting to start a conversation, the observers either did not respond or displayed hostile facial expressions, which was perceived as a lack of empathy [40]. Lees [26] observed that having minimal interpersonal engagements limits the therapeutic potential of interventions, such as formal observation and medications. Patients deprived of interpersonal engagement felt objectified and separated from their health care professionals [26] or that nobody was there for them or acknowledged their existence [40]; these experiences exacerbated their feelings of anxiety and hopelessness and supported their perceptions that nobody cared about them and that their lives were not worth living [40].

#### Control

The “Control” theme involved patients’ need to re-establish a feeling of control over their lives [27]. Suicidal patients experienced a sense of not being in control, a desire to regain control and a sense of losing control during suicidal crises [26, 27], which they often described as periods of overwhelming emotional suffering that left them unable to cope with life [27–31]. Patients whose health care professionals enabled them to *gain insight* and *cope with difficulties and symptoms* were able to regain control of themselves. This sense of control was important for attaining *discharge readiness* and feeling safe from themselves. Patients without this sense of control experienced increased suicidal thoughts.

### Gaining insight

Gaining insight into their illnesses enabled patients to regain control after their suicide attempt [27, 31, 41]; patients who understood themselves were able to address the difficulties in their life without attempting suicide [27] and also felt safer from themselves [31], which helped them feel in control of their lives [41].

### Coping with difficulties and symptoms

Patients felt that a sense of control could be achieved by being able to manage difficulties and by learning new problem-solving and help-seeking skills, as well as by receiving adequate treatment for mental health problems and obtaining assistance for social and economic problems. Patients who were able to manage difficulties were able to visualize a way back to their lives [27, 36, 37]. Variations in coping strategies related to different support and independence needs were described, as some patients expressed a need for others to “fix” their problems, some expressed a need for a break from any type of demand, and others emphasized a need to strengthen their self-efficacy to more effectively cope with their life situations [27, 33, 41]. Some patients experienced that their problems were best addressed through one-on-one conversations with health care professionals [35, 37], whereas others preferred group support [25, 28, 37], spiritual support [25], or family or friend support [25, 38]. Patients needed health care professionals who could adapt to their needs and coping strategies [38].

### Attaining discharge readiness

Patients expressed the expectation that their admission would result in a cure for or solution to their problems; this belief represented a major disconnect between patients’ expectations and the treatment provided during short-term hospitalization [41]. At discharge, some patients felt that their problems were unsolved [37] and that they lacked the skills and tools for coping with their problems and their unchanged circumstances; this feeling resulted in increased distress and suicidal thoughts [41, 42]. At discharge, patients experienced unaddressed problems related to their suicidality [32, 37, 41]. Thus, they did not feel prepared for discharge and feared that leaving the hospital would lead to subsequent suicide attempts [41]. These patients experienced the feeling that the system was failing them and indicated that they did not know where to seek support in the event that formal mental health services could not help [41].

Patients’ sense of control was strengthened by having a post-discharge support plan and by being able to contact the ward after discharge if necessary [27, 35, 41], as well as by being prepared for the upcoming change in their freedom by feeling empowered and supported prior to discharge [31, 41]. Thus, it was important for patients to be allowed to participate in decision making regarding their post-discharge support, as this reduced their fears and anxieties at discharge when being sent “back to the lion’s den” ([41], s. 24).

## Discussion

This paper posed the following review question: “How can we describe suicidal patients’ experiences regarding safety during psychiatric in-patient care?” Suicidal patients’ experiences with safety during psychiatric in-patient care were described in 20 studies that addressed whether their needs were met during their hospitalization. This review argues that patients define safety in terms of “feeling safe” and that connection, protection and control play vital roles in their safety-related experiences. Fulfilment of these needs are experienced as essential for recovery from their suicidal crises, in addition to the ability to feel safe during their encounters with health care professionals and to feel safe from their suicidal impulses. When experiencing unmet needs, the patients not only felt unsafe but also exhibited increased suicidal thoughts and feelings. For some patients, these experiences were characterized as triggers for another suicide attempt.

The patient experiences discussed in our review are related to the relational and emotional aspects of hospital care and are consistent with the findings of other studies regarding patient experiences [10, 13]. Our findings also resonate with those of psychiatric in-patient care studies, in which patients identified psychological safety as the most common safety issue [44]. The *connection* and *protection* components discussed herein emphasize the importance of the therapeutic relationship in not only establishing feelings of safety but also optimizing patient outcomes, such as those related to increases or decreases in patient suicidality. The suicidal patients in this review addressed the vital importance of the therapeutic relationship in helping patients both feel safe and be safe. These findings are consistent with those of studies highlighting the therapeutic alliance in effective suicidal patient assessments and management [45–47] and studies identifying the staff–patient relationship as important to patients’ feelings of safety [44, 48, 49]. Poor staff-patient relationships were found to play key roles in preventable suicides and were attributed to poor communication and relationship quality [50].

This review highlights the importance of addressing the control component to enable suicidal patients to feel and be safe after discharge from the hospital ward. The *control* component demonstrates the importance of supporting external and internal processes that help suicidal patients feel a sense of control and of understanding the individual from an ideographic point of view. Consistent the results of this review, Connell [51] found that, for mental health patients, a sense of control was linked to feelings of safety. The level of desired dependence or independence varied according to each patient’s current circumstances and differed over time.

Undrill [52] stated that psychiatric risks should be perceived as manifestations of suffering. Thus, maintaining high-quality core activities during care and acknowledging suicidal patients’ suffering through trust and therapeutic closeness should be the primary methods of addressing patients’ suicide risk and improving their safety. In accordance with Undrill’s [52] findings, our review indicates that ensuring patient safety entails addressing patients’ therapeutic needs and psychological safety in addition to their physical safety. Although integrating relational and technical patient safety measures into psychiatric care is challenging [53, 54], safety is dependent on this integration. The link between feeling safe and being safe is vital for suicidal patients; suicidal patients’ physical safety cannot be ensured if they do not feel safe. A system that is designed to physically prevent patients from committing suicide but that neglects their need for a connection with health care professionals may not be successful, as patients may exhibit increased suicidality despite the implementation of procedures to prevent this outcome. Furthermore, patients may not only feel unsafe, but they may also be unsafe because of an increased suicide risk imposed by the complex dynamics between emotionally vulnerable patients and their health care professionals. A broader perspective regarding patient safety that integrates therapeutic needs, psychological safety and physical safety is therefore needed.

### Limitations

There were a few limitations to this review. There is a risk of missed studies due to a lack of common nomenclature. To address this limitation, the search terms and strategy were designed to increase the sensitivity to relevant literature. Furthermore, the systematic search included only published peer-reviewed studies, resulting in the exclusion of possibly valuable grey literature and unpublished papers. Although there is a risk of reviewer bias, efforts were made to minimize this bias by applying systematic search methods and by following the PRISMA guidelines for systematic reviews.

The review was limited to studies regarding psychiatric in-patient care. Studies examining the experiences of suicidal patients when receiving emergency care and outpatient treatment were excluded, as were studies regarding the experiences of patients without access to psychiatric care. These types of studies should be included in future reviews that aim to explore patient pathways and continuity of care, as poor continuity of psychiatric care has been associated with preventable suicides [50].

### Implications for research and practice

The literature included a diverse group of patients characterized by suicidal behaviour. These different patient groups may present distinct experiences, thus limiting the general understanding of suicidal patients as a group. To account for the diversity of patients in suicidal crises, more studies involving the elderly, youths, low-income countries and non-Western health care settings are necessary. There is also a need to explore the experiences of suicidal patients in different diagnostic groups, such as suicidal in-patients with/without psychotic symptoms and patients with/without chronic suicidality or borderline personality disorder. The similarities and differences between the experiences of suicidal patients and non-suicidal patients must be elucidated to identify the generic versus group-specific characteristics that determine patient safety in psychiatric care. Additionally, patients may have different needs during different stages of their suicidal crises. For example, Rise et al. [55] observed that patients indicated different safety-related needs depending on their symptoms. However, this distinction was not addressed in the studies included in our review and represents a direction for further research.

We recommend the following changes regarding in-patient care practices for suicidal patients based on the results of our systematic review:

- Patient experiences should be considered an integral part of suicidal patients’ safety to guide clinical practice and the design of patient safety measures.
- Suicidal patients’ need for connection with health care personnel indicate that the relational component of patient safety is considered the most vital aspect of care and should thus be integrated into measures such as constant observation, suicide risk assessments, clinical supervision, ward therapeutic environments and encounters with health care personnel groups.
- Suicidal patients’ need for protection highlights the importance of constant observation in suicidal crises and the need for skilled professionals in close proximity to patients.
- Suicidal patients’ need for control emphasizes the need for therapeutic interventions that increases the patient’s insight and problem-solving skills as well as shared decision making regarding treatment plans, crisis plans, support systems and post-discharge follow-up activities.

## Conclusion

Our review addresses the importance of having a broader view of safety for suicidal patients rather than merely understanding safety in technical terms. When considering suicidal patients’ experiences, safety appears to be related to more than the absence of suicide risk and the need for physical protection. Safety for the suicidal patient is highly dependent on patients’ perceptions of their connections with health care professionals, the fulfilment of their needs during care and their psychological safety. To be safe, patients must feel safe through their *connections* with health care professionals; they must be *protected* against their suicidal impulses and they must have a sense of *control* over their lives. These components should serve as the basis of future efforts designed to understand the ontology of safety for suicidal patients during in-patient psychiatric care.

## Additional files

Additional file 1: Search strategy for PsychINFO. (DOCX 12 kb)
Additional file 2: Table of themes and meaning units. (DOCX 24 kb)
Additional file 3: Malterud’s [22] “Guidelines for authors and reviewers of qualitative studies – an example of checks and scores”. (DOCX 15 kb)
Additional file 4: An overview of the included studies’ scores: high, middle or low. (DOCX 16 kb)
