# Supplementary material for: Suicidal patients’ experiences regarding their safety during psychiatric in-patient care: a systematic review of qualitative studies
Source: BMC Health Serv Res. 2017 Jan 23;17:73. doi: 10.1186/s12913-017-2023-8 (PMC5259991; doi:10.1186/s12913-017-2023-8)
Supplement: Additional file 1: — Search strategy for PsychINFO. (DOCX 12 kb) [file 12913_2017_2023_MOESM1_ESM.docx]

**Search strategy: PsychInfo**

1. patient* experience*
2. inpatient* experience*
3. patient* satisfaction*
4. patient* preference*
5. patient* perception*
6. patient* view*
7. patient* perspective*
8. patient* opinion*
9. user* experience*
10. consumer experience*
11. consumer participation
12. 01 OR 02 OR 03 OR 04 OR 05 OR 06 OR 07 OR 08 OR 09 OR 10 OR 11
13. Suicid*
14. 1 AND 13
15. 2 AND 13
16. 3 AND 13
17. 4 AND 13
18. 5 AND 13
19. 6 AND 13
20. 7 AND 13
21. 8 AND 13
22. 9 AND 13
23. 10 AND 13
24. 11 AND 13
25. 12 AND 13
26. 12.af. AND 13.ab. limiters: peer review,
27. Feeling safe
28. Feeling unsafe
29. 26 AND 28
30. 26 AND 28 limiters peer review.
