# Supplementary material for: Suicidal patients’ experiences regarding their safety during psychiatric in-patient care: a systematic review of qualitative studies
Source: BMC Health Serv Res. 2017 Jan 23;17:73. doi: 10.1186/s12913-017-2023-8 (PMC5259991; doi:10.1186/s12913-017-2023-8)
Supplement: Additional file 2: — Table of themes and meaning units. (DOCX 24 kb) [file 12913_2017_2023_MOESM2_ESM.docx]

|  | **Table of themes and meaning units** | | |
| --- | --- | --- | --- |
|  | **Analytical theme** | **Descriptive themes** | **Meaning units** |
|  | Connection | Meeting someone who cares | 1. Feeling lonely and separated from the external world 2. Expressing a need to be connected to someone who takes time to show that they care 3. Expressing a need to be met by active empathetic listeners 4. Feeling cared for by having their basic needs met 5. Having enough time with providers 6. Feeling cared for when meeting health professionals who exhibit engagement, interest, empathy and hope 7. Feeling valued when meeting someone who cares 8. Feeling as though they have a place in the world by meaning something to others 9. Realizing that they actually do matter reduces suicidal thoughts |
|  |  |  | 1. Experiences that health care professionals do not have time to see patients, and do not care about them 2. Feeling isolated and alone in the ward 3. Feeling stored away, as an object 4. Not having any value as a person 5. Not feeling safe when no one seems to care 6. Lack of interest and engagement 7. Refraining from seeking help and lacking confidence 8. When being isolated and alone in the ward it increases suicidal ideations |
|  |  | Receiving a confirmation of feelings | 1. Expressing a need for someone to understand and confirm their feelings 2. Expressing a need for someone who understands their situation and their story 3. Expressing a need for their suffering to be taken seriously 4. Receiving support regarding their hospitalization 5. Being allowed to express their feelings and talk about their suicidality 6. Receiving confirmation of their feelings enables patients to feel secure and that they have a place in the world 7. Feeling confirmed mitigates feelings of despair 8. Patients feeling understood and confirmed gain insight into and understand their emotions |
|  |  |  | 1. Denying patients their feelings 2. Neglecting patients’ illnesses 3. Focusing on their feelings rather than patients’ feelings 4. Merely discussing patients’ positive resources 5. Not addressing problems that matter 6. Avoiding discussing patients’ suicide attempts 7. Deviating from topics 8. Discussing sensitive issues without confirming them 9. Lack of confirmation results in subsequent suicide attempts |
|  |  | Being acknowledged as a human being | 1. Being met on equal ground as a worthy human being 2. Being treated nonjudgmentally reduces shame 3. Empowerment 4. Being understood as an individual 5. When patients perceive that they are seen as a person, they feel trusted, respected and safe 6. When treated as a human being, patients were receptive to receiving help in the ward 7. Being treated like a worthy equal human being to feel worthy of being alive 8. Feeling respected when involved in decision-making 9. Feeling safe when being seen as a human being 10. Managing to receive help when feeling safe |
|  |  |  | 1. Experiencing distrust when being inspected 2. Feeling unequal and as though they are not perceived as an individual or as though they being punished 3. Feelings of disempowerment when not informed of or included in decision-making 4. Patients who were not seen as equals experienced distrust and did not feel safe 5. Patients who were pre-judged did not feel safe |
|  | Protection | Being protected from death | 1. Expecting health care professionals to protect patients when they are a danger to themselves 2. Being under constant observation prevents suicide and is thus life-saving 3. Feeling safe from suicidal impulses in a protective environment 4. Feeling safe in hospital when safe from suicidal impulses |
|  |  |  | 1. Involuntary hospitalizations increase suffering by not having suicide as an available option 2. Lack of protection in the ward, not feeling safe. 3. Lack of privacy and freedom during constant observation |
|  |  |  |  |
|  |  | Receiving support from observers | 1. A supportive observer is optimistic, acknowledges and empowers individuals and assists with problem solving 2. Internalizing what the observers are signalling 3. Being safe from suicidal impulses trough connections with others 4. Recovery facilitated by receiving observer support |
|  |  |  | 1. Lack of observer support manifests as a lack of empathy and acknowledgement 2. Feeling objectified and detached without observer support 3. Feeling objectified increases stress and hopelessness |
|  | Control | Gaining insight | 1. Need to strengthening a sense of control by gaining insight into their illness 2. Understanding their lives and feelings provides a sense of safety and control |
|  |  |  |  |
|  |  | Coping with difficulties and symptoms | 1. Needing to establish a sense of control to handle difficulties 2. Receiving adequate treatment for mental health problems 3. Receiving help with social and economic problems 4. Needing to improve help-seeking and problem-solving skills 5. Needing others to fix problems 6. A variety of support systems are necessary to meet individual needs |
|  |  | Attaining discharge readiness | 1. Need to understand what to do with their lives (after suicide attempt) 2. Increased sense of security knowing they can contact the ward after discharge 3. The value of post-discharge support provided by health care professionals 4. Need to be empowered regarding discharge planning |
|  |  |  | 1. Expecting to receive help or a cure in the hospital, a disconnect between expectations and treatment provided in the hospital 2. Not addressing the problems that matter during hospitalization 3. Experiencing additional problems after discharge 4. Lacking the tools for coping at discharge 5. Anxiety at discharge caused by leaving a place of safety 6. What to do with their lives after discharge 7. Patients not receiving expected help believe that the system has failed them down 8. Unsolved problems and a lack of coping skills increase distress and suicidal thoughts at discharge |
|  |  |  |  |
