# Supplementary material for: Suicidal patients’ experiences regarding their safety during psychiatric in-patient care: a systematic review of qualitative studies
Source: BMC Health Serv Res. 2017 Jan 23;17:73. doi: 10.1186/s12913-017-2023-8 (PMC5259991; doi:10.1186/s12913-017-2023-8)
Supplement: Additional file 3: — Malterud’s [22] “Guidelines for authors and reviewers of qualitative studies – an example of checks and scores”. (DOCX 15 kb) [file 12913_2017_2023_MOESM3_ESM.docx]

**Malterud’s (2001) Guidelines for authors and reviewers of qualitative studies – an example of checks and scores.**

| Example: Cardell & Pitula (1999). Suicidal inpatients' perceptions of therapeutic and nontherapeutic aspects of constant observation | |
| --- | --- |
| 1. Aim    1. *Is the research question a relevant issue?*    2. *Is the aim sufficiently focused, and stated clearly?*    3. *Does the title of the article give a clear account of the aim?* | **Middle:** No research question, not a clearly stated aim, but the purpose of the study and title is clearly presented and relevant for the study |
| 1. Reflexivity    1. *Are the researcher's motives, background, perspectives, and preliminary hypotheses presented, and is the effect of these issues sufficiently dealt with?* | **Low:** The authors’ background or pre-understanding is not presented, and its effect is not dealt with. |
| 1. Method and design    1. *Are qualitative research methods suitable for exploration of the research question?*    2. *Has the best method been chosen with respect to the research question?* | **High:** The method is in line with the research question. |
| 1. Data collection and sampling    1. *Is the strategy for data collection clearly stated (usually purposive or theoretical, usually not random or representative)?*    2. *Are the reasons for this choice stated?*    3. *Has the best approach been chosen, in view of the research question?*    4. *Are the consequences of the chosen strategy discussed and compared with other options?*    5. *Are the characteristics of the sample presented in enough depth to understand the study site and context?* | **Middle:** The sample characteristics and inclusion criteria’s are described, but the reasons for the choice is not stated. A discussion of other options is missing. |
| 1. Theoretical framework    1. *Are the perspectives and ideas used for data interpretation presented?*    2. *Is the framework adequate, in view of the aim of the study?*    3. *Does the author account for the role given to the theoretical framework during analysis?* | **Low:** A reference is given to grounded theory, but the role given during analysis is not stated. Perspectives not presented. |
| 1. Analysis    1. *Are the principles and procedures for data organization and analysis fully described, allowing the reader to understand what happened to the raw material to arrive at the results?*    2. *Were the various categories identified from theory or preconceptions in advance, or were they developed from the data?*    3. *Which principles were followed to organise the presentation of the findings?*    4. *Are strategies used to validate results presented, such as cross-checks for rivalling explanations, member checks, or triangulation. If such strategies are not described in this section, they should appear as validity discussions later in the report.* | **Low:** No procedures for data analysis is presented. Hutchinsons’ recommendation for managing data is followed, but not explained. No validation strategies. |
| 1. Findings    1. *Are the findings relevant with respect to the aim of the study?*    2. *Do they provide new insight?*    3. *Is the presentation of the findings well organised and best suited to ensure that findings are drawn from systematic analysis of material, rather than from preconceptions?*    4. *Are quotes used adequately to support and enrich the researcher's synopsis of the patterns identified by systematic analysis?* | **High:** The findings are relevant, they provide new insight, they are well organized and adequate quotes are presented. |
| 1. Discussion    1. *Are questions about internal validity (what the study is actually about), external validity (to what other settings the findings or notions can be applied), and reflexivity (the effects of the researcher on processes, interpretations, findings, and conclusions) addressed?*    2. *Has the design been scrutinised?*    3. *Are the shortcomings accounted for and discussed, without denying the responsibility of choices taken?*    4. *Have the findings been compared with appropriate theoretical and empirical references?*    5. *Are a few clear consequences of the study proposed?* | **Middle: T**he discussion is in line with the research question and is compared with previous research. The conclusion section provides some clear consequences. However, discussions about validity is lacking. |
| 1. Presentation    1. *Is the report easy to understand and clearly contextualised?*    2. *Is it possible to distinguish between the voices of the informants and those of the researcher?* | **High:** The report is easy to read and understand. |
| 1. References    1. *Are important and specific sources in the field covered, and have they been appropriately presented and applied in the text?* | **High:** Important literature is presented in the test. |
