# Supplementary material for: Suicidal patients’ experiences regarding their safety during psychiatric in-patient care: a systematic review of qualitative studies
Source: BMC Health Serv Res. 2017 Jan 23;17:73. doi: 10.1186/s12913-017-2023-8 (PMC5259991; doi:10.1186/s12913-017-2023-8)
Supplement: Additional file 4: — An overview of the included studies’ scores: high, middle or low. (DOCX 16 kb) [file 12913_2017_2023_MOESM4_ESM.docx]

**An overview of the included papers’ scores: high, middle or low.**

| **Author(s)** | | **1.**  **Aim** | **2. Reflexivity** | **3. Method and design** | **4.**  **Data collection and sampling** | **5. Theoretical framework** | **6. Analysis** | **7. Findings** | **8. Discussion** | **9. Presentation** | **10. References** |
| --- | --- | --- | --- | --- | --- | --- | --- | --- | --- | --- | --- |
| *1* | *Vatne & Nåden, 2016* | high | high | high | high | high | high | high | middle | high | high |
| *2* | *Lees, Procter and Fassett 2014.* | high | middle | high | high | middle | high | high | middle | high | high |
| *3* | *Montross Thomas et al. 2014.* | middle | low | high | high | low | middle | high | high | high | high |
| *4* | *Vatne & Nåden, 2014.* | high | high | high | high | high | high | high | high | high | high |
| *5* | *Cutcliffe et al. 2012a.* | high | high | high | high | middle | high | high | middle | high | high |
| *6* | *Cutcliffe et al. 2012b.* | high | high | high | high | middle | high | high | middle | high | high |
| *7* | *Pavulans, Blomsjo, Edberg & Oljehagen et al. 2012.* | high | high | high | high | middle | high | high | high | high | high |
| *8* | *Vatne & Nåden, 2012.* | high | middle | high | high | high | high | high | high | high | high |
| *9* | *Holm &, Severinsson, 2011.* | high | middle | high | high | high | high | high | high | high | high |
| *10* | *Cutcliffe Stevenson, Jackson & Smith, 2006.* | high | middle | high | high | high | high | high | middle | high | high |
| *11* | *Sun et al. 2006b.* | high | low | high | high | middle | high | middle | high | high | high |
| *12* | *Sun et al. 2006a.* | high | low | high | high | middle | low | middle | middle | high | high |
| *13* | *Talseth, Gilje & Nordberg, 2003.* | high | middle | high | middle | high | high | high | middle | high | high |
| *14* | *Wiklander, Samuelsson, & Åsberg, 2003.* | high | high | high | high | middle | high | middle | middle | high | high |
| *15* | *Talseth, Jacobsson & Nordberg, 2001.* | high | high | high | high | high | high | high | high | high | high |
| *16* | *Samuelsson et al., 2000.* | high | middle | high | high | middle | high | high | high | high | high |
| *17* | *Cardell & Pitula, 1999.* | middle | low | high | middle | low | low | high | middle | high | high |
| *18* | *Fletcher, 1999.* | high/middle | low | low | low | low | high | low | low | low | middle |
| *19* | *McLaughlin, 1999.* | high | middle | low | low | middle | middle | middle | middle | middle | high |
| *20* | *Talseth et al., 1999.* | high | low | high | high | high | high | high | high | high | high |
